# Designing Collagen-Binding Peptide with Enhanced Properties Using Hydropathic Free Energy Predictions

**DOI:** 10.3390/app13053342

**Published:** 2023-03-06

**Authors:** Kyle Boone, Aya Kirahm Cloyd, Emina Derakovic, Paulette Spencer, Candan Tamerler

**Affiliations:** 1 Institute for Bioengineering Research, University of Kansas, 5109 Learned Hall 1530 W, 15th Street, Lawrence, KS 66045-7609, USA; 2 Department of Mechanical Engineering, University of Kansas, Lawrence, KS 66045-7609, USA; 3 Bioengineering Program, University of Kansas, 1132 Learned Hall 1530 W, 15th Street, Lawrence, KS 66045-7609, USA

**Keywords:** collagen, peptide design, binding free energy, peptide-protein

## Abstract

Collagen is fundamental to a vast diversity of health functions and potential therapeutics. Short peptides targeting collagen are attractive for designing modular systems for site-specific delivery of bioactive agents. Characterization of peptide–protein binding involves a larger number of potential interactions that require screening methods to target physiological conditions. We build a hydropathy-based free energy estimation tool which allows quick evaluation of peptides binding to collagen. Previous studies showed that pH plays a significant role in collagen structure and stability. Our design tool enables probing peptides for their collagen-binding property across multiple pH conditions. We explored binding features of currently known collagen-binding peptides, collagen type I alpha chain 2 sense peptide (TKKTLRT) and decorin LRR-10 (LRELHLNNN). Based on these analyzes, we engineered a collagen-binding peptide with enhanced properties across a large pH range in contrast to LRR-10 pH dependence. To validate our predictions, we used a quantum-dots-based binding assay to compare the coverage of the peptides on type I collagen. The predicted peptide resulted in improved collagen binding. Hydropathy of the peptide–protein pair is a promising approach to finding compatible pairings with minimal use of computational resources, and our method allows for quick evaluation of peptides for binding to other proteins. Overall, the free-energy-based tool provides an alternative computational screening approach that impacts protein interaction search methods.

## 1. Introduction

Collagen type I (COL1) is among the list of the most abundant fibrous proteins in the human body. It contributes to the mechanical stability, strength, and toughness of a range of tissues including dentin, bone, skin, and ligaments [1]. Collagen matrices are important components of the extracellular matrix (ECM), as they provide structural support to the extracellular space of connective tissues. As a complex biopolymer network, the ECM accommodates several biochemical interactions and impacts the physical environment for various physiological processes including pathological and healing states. Collagen matrices are frequently used in biomaterials that are developed for tissue engineering applications [2,3]. Among the 28 subtypes of collagen identified to date, 90% of collagen in the human body is COL1 [3]. Among the many interacting partners with collagen are extracellular matrix components [4,5], decorin [6,7], and other leucine-rich repeat (LRR) proteins [8]. Considering the wide range of interactions and impact on the tissues, functionalization of collagen and collagen mimetic fibrillar self-assemblies with bioactive agents have been the subject of intense investigations. These investigations have explored a range of applications including regenerative medicine to target delivery of therapeutics and tailoring biomaterials for mechanical properties. Short peptides are particularly attractive bioactive agents because of their flexibility and binding affinity when targeting complex biopolymer and hybrid interfaces [9–11]. Furthermore, targeting peptides through the incorporation of training methods allows for including any desired bioactivity domain into the site [12–17]. Yet, there are a limited number of large data sets for peptide sequences that specifically interact with target proteins or composite systems [18].

Collagen is a protein supramolecular assembly of tropo-collagen fibrils whose dynamic inter-chain interactions respond to changes in pH, temperature, and solute concentrations [5,19,20]. Some conditions, such as pH and temperature, at the molecular scale have been investigated to determine their impact on the structural state of collagen from molecular dynamics simulations [21–23]. Collagen’s supramolecular structure compels designing distinct interactions for peptides from other types of proteins. Helical formations of collagen can enable shorter sequence conjunctions than is possible with a single polypeptide strand. The triple helix structure weaves three separate chains composed of two alpha chain 1 (COL1A1) and one alpha chain 2 (COL1A2) such that the contact residues depend on the stagger between the chains and the corresponding overlapping residues. This interaction was recently studied in a collagen-mimetic system, which showed that the specific amino acid sequence may have less influence on the fibril formation than initially thought [24]. Computational studies of collagen-mimetic systems gave further understanding of the structural organization [21,25] including proposing interactions with calcium phosphate minerals [26].

Decoding the logic contained within the biophysics of peptide–protein interactions is a challenge underlying both the treatment of disease states relating to collagen and the potential source of several collagen mimetic applications. Recent machine learning (ML) advances have created deep learning neural networks for peptide–protein interactions [27]. While many interactions are successfully identified with deep learning models, a limitation of such models is the variable prediction performance of untrained cases, which are necessarily used in generative tasks. New regularization methods are being developed to address this limitation. [28,29]. Physics-based approaches are recognized for further fostering regulation of ML techniques as a screen in classification or generation tasks [30,31].

Characterizing peptide–protein binding interactions involves matching two polypeptide chains concurrently. Molecular docking is a method for computationally matching structural interfaces. The molecular docking search space is high dimensional, as selections are made for twelve degrees of freedom: three axes of rotation, three axes of translation, and two interaction partners. Because of the large number of potential interactions, screening methods are necessary to identify promising peptide–protein combinations prior to evaluation. In predicting these interactions, bioavailability of the peptides becomes critical to promote an effective binding interface. Since water solubility will improve bioavailability, hydropathy of the peptide–protein pair is considered as a promising approach to finding compatible pairings with the minimal use of computational resources [18,32].

Sense and antisense peptides are more likely to interact based on hydropathy complementation in the genetic code, as recognized by Mekler [33,34]. Hydropathy is recognized as a physical property that is predictive of peptide–protein interactions based upon hydrophilicity and hydrophobicity properties [35]. De Souza and Brentani demonstrated that a sense peptide for human collagen (type I alpha chain 2), which binds to type I collagen, has a similar hydropathy plot as a collagenase-derived collagen-binding peptide, even with low sequence homology between the peptide sequences [36]. We are inspired by the concept of sense–antisense peptide–protein interactions by formulating a light-weight screening method.

In this work, we develop a screening method that uses the hydropathy-derived free energy scales of the amino acids to create features of adjacent intra-chain residues to score the single-site binding energies of any peptide–collagen interaction. The screening method predicts the free energy of binding between any consecutive three amino acids in the peptide to any consecutive three amino acids on the collagen chain. We use the Wimley–White hydropathy scale [37–39] of the experimentally determined free energies of peptides to estimate the hydropathy of each residue by its side chain interactions. The rules for combining the side chain free energies resulting in a non-covalent bond are selected to model primarily electrostatic interactions. Since the Wimley–White hydropathy scale explicitly includes distinct charge states for acidic and basic amino acid side chains, our method also explicitly accounts for pH states of amino acid side chains. We initiated our search using a collagen-binding domain analogue that was derived from the sense peptide TKKTLRT for collagen type I α(2) [36]. After comparing the most probable binding sites of TKKTLRT and a collagenase collagen-binding peptide, we provide a first-order approximation of the free energy of binding for well-studied collagen-binding peptides and include a hydrophobic binding site building upon pH stability. We show how the predicted binding varies across collagen type I alpha chain 2 at four different pH levels: 2, 4, 7, and 9. Finally we experimentally validated our designed peptide and demonstrated the enhanced peptide coverage on type I collagen.

## 2. Materials and Methods

### 2.1. Potential Free Energy

The free energy estimates were based on differences in average hydropathy for corresponding neighboring residues between two polypeptide chains. The average hydropathy is the sum of the Wimley–White hydropathy scale value [40] for the free energy difference of solvation in water compared to solvation in octanol for the current residue and its neighbors. While the hydropathy scale has been used to study membrane folding dynamics for proteins, the hydropathy difference between solubility in water and solubility in octanol is more generally applicable if the first-order approximation is made that octanol is idealized as a completely non-polar molecule. If this approximation is made, effects in which the heterogeneity of the charge within octanol’s volume are ignored. This assumption simplifies the classification of hydropathy phases.

#### 2.1.1. Relatively Homogeneous Electron Density Interface

Negative hydropathy values are hydrophobic regions of space with roughly homogeneous electron density. The higher the hydropathy value, the more homogeneous the electron density is. When both peptide plots match having a negative hydropathy value, the free energy assigned to the interaction is additive, implying that a paired interaction is more favorable than either component having the interface replaced with water. The potential energy is assigned as the sum of the magnitudes of the residues. This calculation assumes that the interface of the hydrophobic residues and water is similar to the interface of octanol and water to a first-order approximation.

#### 2.1.2. Relatively Heterogeneous Electron Density Interface

For positive hydropathy values, there are two regimes. The first is that one residue has a relatively high electron density (electro-negative), and the other residue has a relatively low electron density (electro-positive). When the residues interact, they form a more homogeneous electron density distribution, which is closer to the octanol interface than water interface compared to before the interaction. This is a favorable free energy interaction. The magnitude of the interaction is limited to the homogeneity possible by the minimum electronegativity difference of the two differences. The resulting potential is the negative of the minimum hydropathy value.

The second regime is that each residue has the same heterogeneous electron density, and combining the two electron densities results in a larger heterogeneous electron density distribution. This is not favored by the polarized electron density of the solvent because the solvent can create more homogeneous electron density if the pairing is between solvent and amino acid residues instead of between the amino acid residues themselves. Because the solvent molecule is by far the majority, the entropy of the system predicts that solvent molecules will absorb the unbalanced electron density compared to the other residues of heterogeneous charge. Therefore, this kind of interaction is assigned the free energy as the positive of the minimum hydropathy value.

#### 2.1.3. Relatively Homogeneous Electron Density and Relatively Heterogeneous Electron Density Interface

If one interface has negative hydropathy and the other has positive hydropathy, then there is heterogeneous electron density for the positive hydropathy side which is not balanced by the negative hydropathy side. This is an unfavorable interaction, as the polarity of the water can create more homogeneous electron density than the other interfacial partner. This interaction is assigned the bonding free energy of the positive of the minimum hydropathy value.

#### 2.1.4. Summary Equations

The above potential free energy interactions are quantifiable. The equations below cover the different interaction cases of homogeneous or heterogeneous electron density interactions.

(1) $$\text{if}\;{\hat{\Delta G_{woct\;}}}^{collagen\;}<0\;AND\;{\hat{\Delta G_{woct\;}}}^{peptide}<0,\;\text{then}$$

$$\Delta G_{single-site\;interaction\;}=-sum\left(abs\left({\hat{\Delta G_{woct\;}}}^{collagen\;}\right),abs\left({\hat{\Delta G_{woct\;}}}^{peptide}\right)\right)$$

(2) $$\text{else}\;\text{if}\;{\hat{\Delta G_{woct\;}}}^{collagen\;}<0\;XOR\;{\hat{\Delta G_{woct\;}}}^{peptide}<0,\;\text{then}$$

$$\Delta G_{single-site\;interaction\;}=min\left(abs\left({\hat{\Delta G_{woct\;}}}^{collagen\;}\;\right),abs\left({\hat{\Delta G_{woct\;}}}^{peptide}\right)\right)$$

(3) $$\text{else}\;\text{if}\;{\hat{\Delta G_{woct\;}}}^{collagen\;}>0\;AND\;{\hat{\Delta G_{woct\;}}}^{peptide}>0,\;\text{then}$$

If collagen electronegativity > 0 XOR peptide electronegativity > 0, then

$$\Delta G_{single-site\;interaction\;}=\;-min\left(abs\left({\hat{\Delta G_{woct\;}}}^{collagen\;}\right),abs\left({\hat{\Delta G_{woct\;}}}^{peptide}\right)\right)+\;\;\left(max\left(\hat{\Delta G_{woct\;}}\right)-min\left(\hat{\Delta G_{woct\;}}\right)\right)$$

else

$$\Delta G_{single-site\;interaction\;}=abs\left({\hat{\Delta G_{woct\;}}}^{collagen\;}\right)+abs\left({\hat{\Delta G_{woct\;}}}^{peptide}\right)$$

### 2.2. Imaging Collagen-Binding Peptides with Quantum Dots on Rat-Tail Type I Collagen

#### 2.2.1. Peptide Synthesis

TKKTLRT and TKKLTLRT peptides were synthesized on RINK amide resin (RFT105, AAPPTEC, Louisville, KY, USA) using the AAPPTEC Focus XC synthesizer (Louisville, KY, USA) with standard Fmoc solid-phase peptide synthesis protocol. Post-synthesis, the peptides were biotinylated, taking advantage of avidin–biotin chemistry for running collagen-binding assays using streptavidin-conjugated quantum dots. Peptide biotinylation followed the AAPPtec protocol for activation and coupling of D-biotin (AAPPTEC, Louisville, KY, USA). The peptide was suspended in dimethylformamide (DMF, 9.8%, Sigma-Aldrich, St. Louis, MO, USA) at a volume of 10 mL per gram of resin-bound peptide. Separately, 1 mmol of biotin per 1 mL solution of dimethyl sulfoxide (DMSO, 99.7%, Sigma-Aldrich, St. Louis, MO, USA) and DMF (1:1 *v*/*v*) is prepared. One equivalent of hexafluorophosphate (HBTU, AAPPTEC, Louisville, KY, USA) and *N*,*N*′-Diisopropylethylamine (DIEA, 99.5% nitrogen flushed, Acros Organics, Fair Lawn, NJ, USA), respectively, were added to the mixture and stirred at room temperature until the biotin was fully dissolved. Once dissolved, the resin-bound peptide solution was added and mixed at room temperature for three hours. After biotinylation was complete, the peptides were filtered, washed with ethanol, dried, and ready for cleavage. Peptide cleavage followed the AAPPTEC protocol. The cleavage cocktail of the peptides contained trifluoroacetic acid (TFA, 99%, Sigma-Aldrich, St. Louis, MO, USA), thioanisole (99%, Sigma-Aldrich, St. Louis, MO, USA), 1,2-ethanedithiol (95%, Acros Organics, Fair Lawn, NJ, USA), triisopropylsilane (98%, Sigma-Aldrich, St. Louis, MO, USA), and DI water at the following ratios: 87.5:5.0:2.5:2.5:2.5. The peptides were left in the cleavage cocktail on a rotator for 2 h, subsequently precipitated in cold ether, and lyophilized.

Peptide purification was initially performed on the semi-preparative reverse-phase high-pressure liquid chromatography (HPLC) Waters system, with the Waters 600 controller and Waters 2487 Dual Absorbance Detector. The system used the 10 μm C-18 silica Luna column (250 × 10 mm, Phenomenex Inc., Torrance, CA, USA). The mobile phase was comprised of phase A (94.5% HPLC grade water, 5% acetonitrile, 0.5% TFA) and phase B (100% acetonitrile). Lyophilized peptides were dissolved in 4 mL of phase A then purified on a linear gradient at 0.5% phase B·min^−1^ (5%–85% phase B) with a flow rate of 3 mL·min^−1^ with detection at 254 nm, which was conducted at room temperature. The resulting purified peptide fractions collected were verified using the analytical Shimadzu HPLC system, an LC-2010 HT liquid chromatograph, and an SPD-M20A prominence diode array detector. A 5 μm C-18 silica Luna column (250 × 4.6 mm, Phenomenex Inc., Torrance, CA, USA) was used with a mobile phase composed of phase A (99.9% HPLC-grade water, 0.1% TFA) and phase B (100% acetonitrile). The system ran on a linear gradient with 1 mL·min^−1^ flow, 40 °C, and 254 nm detection. The purified peptides were lyophilized and stored at −20 °C.

#### 2.2.2. Collagen–Peptide Sample Preparation

Round mica with a diameter of 12 mm (Grade V1, 0.21 mm thickness from Ted Pella Inc., Redding, CA, USA) was used as the substrate surface. A total of 50 μM peptide (solute) in type 1 rat tail collagen suspension (solvent, 3 mg/mL, C3867 from Sigma-Aldrich, St. Louis, MO, USA) was prepared by gently dissolving and mixing lyophilized peptide into the collagen suspension. A total of 150 μL of the peptide-suspension was drop-casted on the mica, left to self-assemble at 4 °C, and further examined after it was dry (approximately 16 h).

#### 2.2.3. Quantum Dot Binding Assay

The binding capability of the TKKTLRT and TKKLTLRT peptides to collagen were evaluated using streptavidin-conjugated Quantum dots (Q-dots) with emission wavelength at 655 nm from Thermo Fisher Scientific (Vilnius, Lithuania). Collagen-peptide surfaces and collagen were mounted individually on precleaned plain microscope slides (Fisher Scientific, Fair Lawn, NJ, USA). A total of 30 μL of a 5 nM Q-dot solution (in DI water) was drop-casted on the collagen–peptide surfaces and collagen control and left to incubate at room temperature, protected from light, until dry. The surfaces were subsequently washed in dark conditions with 0.35 mL of DI water from a transfer pipette slowly dropped on the surface of the sample at an angle to allow the drops to roll off the surface. The samples were allowed to dry before being washed a second time and allowed to dry again. Drying occurred in the same conditions as incubation. The samples were stored in a desiccator at 4 °C prior to imaging.

#### 2.2.4. Confocal Microscopy

Confocal microscopic images were taken of the collagen–peptide surfaces treated with Q-dots at 10X (Leica ACS APO/0.30 CS) and 20X (Olympus UPlanSApo/0.75 with M25 × 0.75 threaded ThorLabs adapter, Cat #RMSA1) magnification in air on a Leica TCS SPE Laser Scanning upright microscope (Model DMR-Q, Leica Microsystems, Wetzlar, Germany), courtesy of the Microscopy and Analytical Imaging Research Resource Core Laboratory (RRID:SCR_020231). Q-dot fluorescence was captured as a Z-stack, per magnification level, at a laser intensity of 15% with an excitation wavelength of 405 nm and an emission wavelength of 655 nm (643 nm to 670 nm excitation range was evaluated), as indicated per ThermoFisher Scientific. Z-stack slices were of 12-bit-depth and obtained at a 2048 × 2048 resolution with a 360 ns pixel dwell time and at a 0.185/s frame rate. Additionally, images were 550 × 550 um in size with a 268.29 × 268.69 nm pixel size and 75.54 μm pinhole size over a 2.057 μm optical section.

#### 2.2.5. Fluorescent Image Processing

Images were pre-processed using ImageJ 1.53 t [41]. They underwent a Z-projection at maximum intensity. A median filter was then applied at a two-pixel radius and were subsequently normalized with contrast enhancement at 0.15%. Slices at the lower and higher regions of the Z-stacks were evaluated for elimination based on their contribution to over-saturation and speckle noise that would affect image coherence and spatial resolution.

## 3. Results

### 3.1. Potential Binding Sites for Collagen Type I Subsequence

A recent study on fibromodulin interaction with collagen and activation of lysyl oxidase tested the ability of collagen III sequences to bind to collagen I [42]. A binding motif from part of the study was found to be GLKGHR. The study further showed which parts of the motif bind to the collagen by showing that a similar peptide GLAGHA binds significantly less than GLKGHR. The free-binding energies from our method are consistent with these results and predict binding behaviors for alkaline (pH 9) and acidic (pH 2) conditions. We verified that our hydropathy-based free-energy-binding screening method correctly predicted the result of this known study. See Figure S1.

**Figure 1.**
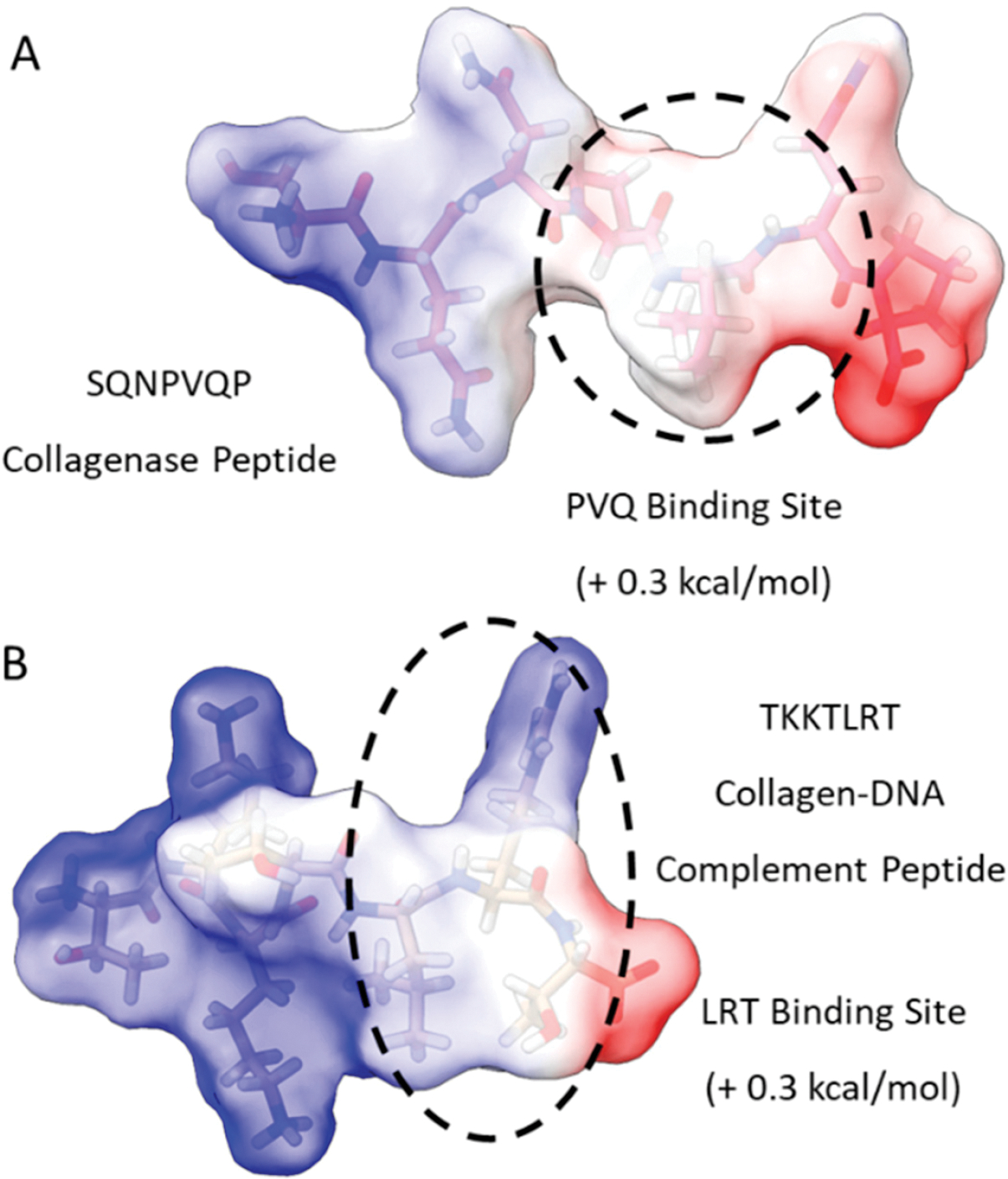
Free energy of binding estimates for sets of three adjacent residues for two different collagen-binding peptides (collagenase peptide (**A**) and collagen DNA-complement peptide (**B**)) on human collagen type I alpha chain 2 (COL1A2) residues 861–871. Each circle represents a potential three-amino-acid binding site between a peptide and collagen as a result of the lowest predicted energy binding subsequence for each respective peptide. The potential binding free energy was calculated from the Wimley–White hydropathy scale values for the amino acids within the binding site.

We initiated our search using a collagen-binding domain analogue (TKKLTLRT) that was derived from the sense peptide TKKTLRT for collagen type I α(2) [36]. This peptide was engineered by a rational hydropathy feature inspired by a leucine-rich repeat (LRR) within the decorin glycoprotein. We first compared the most probable binding sites of TKKTLRT and a collagenase collagen-binding peptide (SQNPVQP) (Figure 1). To find the binding sites, we provided a first-order approximation of the free energy of binding for well-studied collagen-binding peptides TKKTLRT, SQNPVQP, and LRR-10 (leucine-rich repeat-10) to collagen type I [10]. Then we included a more pH-stable version of the hydrophobic binding site in LRR-10 (LRE LHL NNN) into an analogue of TKKTLRT by inserting a leucine residue (TKK LTL RT) and predicted the peptide binding at different pH levels.

Our screening tool is based on the Wimley–White scale, which uses measured free energy differences in solvation among water, octanol, and POPC (bilayer membrane molecule) of penta-peptides to infer the free energy changes of each of the side chains of the canonical amino acids. Our screening method focuses on the free energy difference between water and octanol, with octanol being considered as completely hydrophobic for our first approximation. Our screening tool uses the electrostatic interaction rules of electronegativity to determine the difference between attractive and repulsive interfaces. We averaged the hydropathy in segments of three amino-acid sub-sequences.

**Figure 2.**
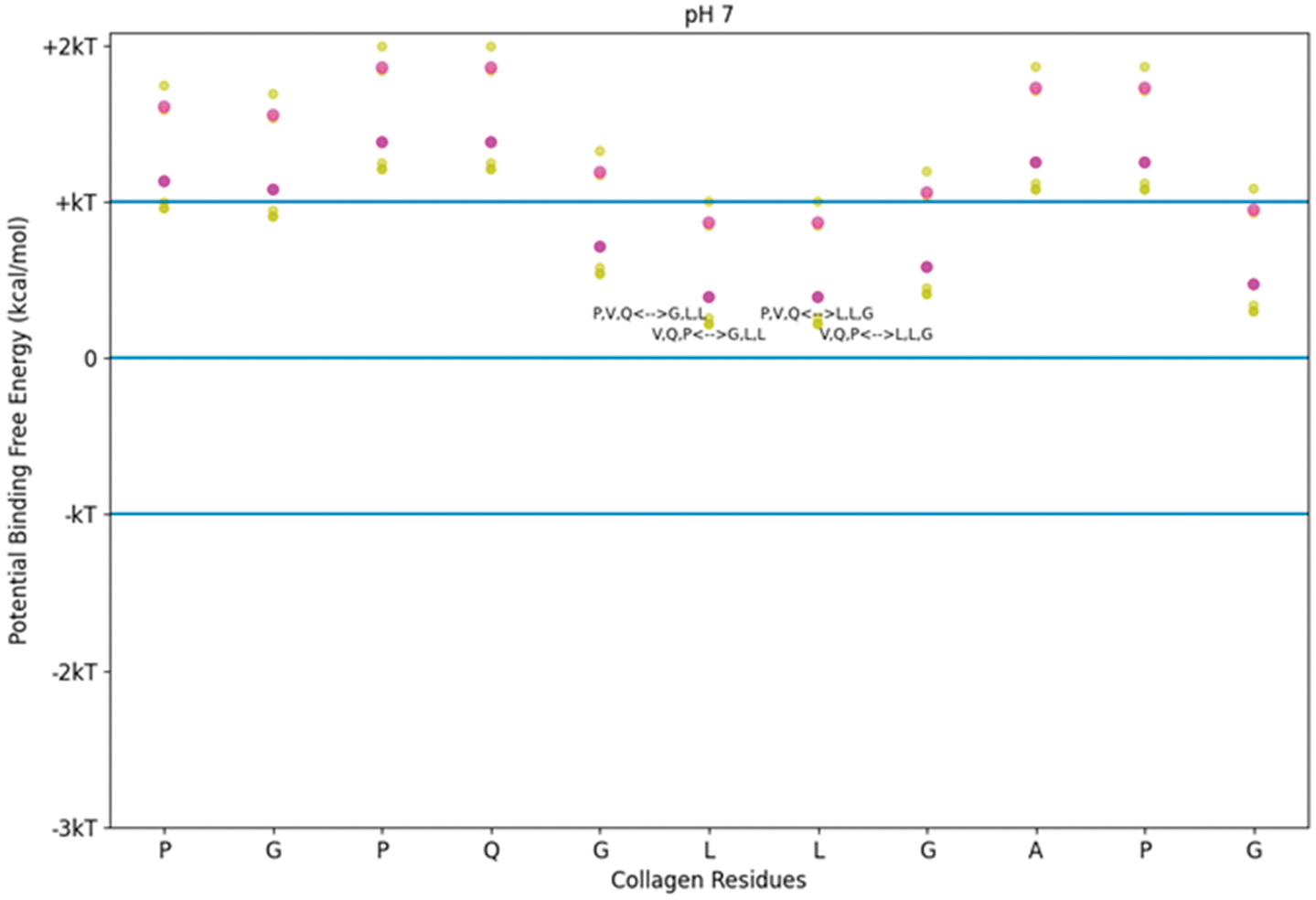
Free energy of binding estimates for sets of three adjacent residues on two different collagen-binding peptides on human collagen type I alpha chain 2 (COL1A2) residues 861–871. Positive binding sites are energetically unfavorable, while negative binding sites are favorable. Binding sites significantly below −kT are likely to be stable. Each circle represents a potential three-amino-acid binding site between a peptide and collagen. The potential binding free energy was calculated from the Wimley–White hydropathy scale values for the amino acids within the binding site. The three-amino-acid binding sites are labeled with the peptide sub-sequence when the potential energy was below + 0.3 kcal/mol. Potential binding sites are gold for collagenase peptide (SQNPVQP) and magenta for the DNA complement peptide (TKKTLRT). The radius of the circle sizes is related to the magnitude of the polarity of the peptide residues. kT, which is the Boltzmann’s constant multiplied by the temperature of 25 °C, is 0.693 kcal/mol. This value is close to a mean estimate of the free energy fluctuations of a surrounding environment near room temperature. The temperature dependence is based on the absolute temperature, so body temperature would have a corresponding kT of about 0.721 kcal/mol (about 4% increase).

Using the screening tool, we determined the predicted free energies of binding between TKKTLRT and the collagen sub-sequence for which TKKTLRT is the DNA complement. We also included SQNPVQP as a near match for the hydrophobicity trend in TKKTLRT. We observed that while all potential binding energies using this method were positive, some were near zero (See Figure 2). Zero potential free energy means that the process may happen if another reaction releasing energy takes place nearby. The normal fluctuation of free energy is described by the product of the Boltzmann’s constant and the absolute temperature of the system. For 25 °C, the normal fluctuation is +/− 0.693 kcal/mol, referred to as kT. For body temperature at 37 °C, the kT value is about 4% larger (0.721 kcal/mol). The kT values in the figures for this study use 25 °C, though which kT levels apply between the room temperature value and the body temperature value are close to the same. While the interaction between TKKTLRT and its antisense collagen sequence is not favored according to this approximation, the interaction is still allowable. The approximation also implies that the interaction between either TKKTLRT or SQNPVQP and collagen at this site is highly reversible. Stronger hydrophobicity interactions are possible if we consider sequences which are not between sense/antisense pairs.

**Figure 3.**
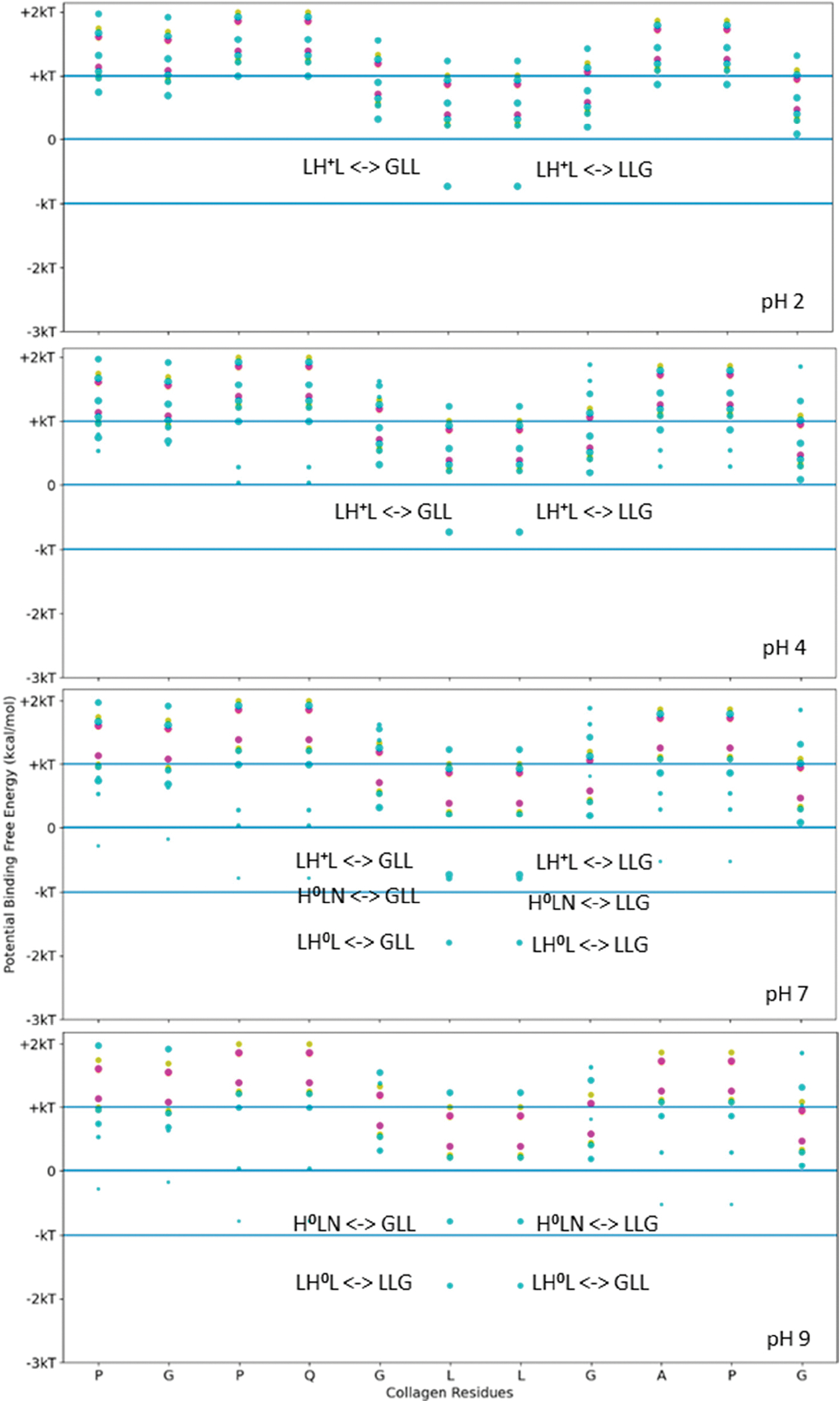
Free energy of binding estimates for sets of three adjacent residues for different collagen-binding peptides on human collagen type I alpha chain 2 (COL1A2) residues 861–871. Positive binding sites are energetically unfavorable, while negative binding sites are favorable. Binding sites significantly below −kT are likely to be stable. Each circle represents a potential three-amino-acid binding site between a peptide and collagen. The potential binding free energy was calculated from the Wimley–White hydropathy scale values for the amino acids within the binding site. The three-amino-acid binding sites are labeled with the peptide sub-sequence when the potential free energy is below −0.693 kcal/mol. When the histidine is protonated, the free energy shifts from −1.2 kcal/mol to −0.5 kcal/mol. Potential binding sites are gold for collagenase sub-sequence (SQNPVQP), cyan for LRR-10 (LRELHLNNN), and magenta for the COL1A2 sense peptide (TKKTLRT). The radius of the circle sizes is related to the magnitude of the polarity of the peptide residues. The middle leucine residues of the COL1A2 sub-sequence mark the potential free energy change of binding when the histidine in the LRR-10 sequence transitions from uncharged to protonated. The potential energy of binding at the GLL/LLG collagen site is similar for the protonated histidine site and the non-protonated histidine site with the asparagine residue (H0-L-N, a shift of one amino acid residue for LRR-10 from L-H0-L). kT, which is the Boltzmann’s constant multiplied by the temperature of 25 °C, is 0.693 kcal/mol. This value is close to a mean estimate of the free energy fluctuations of a surrounding environment near room temperature.

Functional applications that label collagen, however, are more reliable if the stability of the fusion–tag interface to collagen is based upon a robust and stable interaction. For higher-stability binding interactions, we considered a stronger binding example to pattern our hydropathy relationship. LRR-10 is a decorin subunit shown to bind with high affinity and high stability to type I collagen when used within fusion constructs. We used the above scanning tool to identify likely single-binding sites between LRR-10 and the same collagen subunit. We calculated the potential free energy of binding for sets of three adjacent residues for different collagen-binding peptides on human collagen type I alpha chain 2 (COL1A2) residues at different pH levels ranging from acidic to basic (Figure 3). Figure 3 shows that LRR-10 has many more hydropathy-favored interactions predicted for the COL1A2 sub-sequence considered in Figure 2 compared to either SQNPVQP or TKKTLRT. The explicit hydropathy shift provided by the screening method highlights the contribution of the histidine in the LRR-10 sequence to maximizing the electrostatic interactions between the L-H0-L tripeptide of LRR-10 and the G-L-L/L-L-G tripeptides of the COL1A2 sub-sequence in pH 7 and pH 9 environments. The middle leucine residues of the COL1A2 sub-sequence mark the potential free energy change of binding when the histidine in the LRR-10 sequence transitions from uncharged to protonated. In lower pH environments, the L-H + -L tripeptide is too polar to be an electrostatic fit below the −kT energy level. As the conjugate base of histidine, the L-H0-L tripeptide is a better electrostatic fit for the COL1A2 sub-sequence, with the predicted interaction favored near the −2 kT level. The potential energy of binding at the GLL/LLG collagen site is similar for the protonated histidine site and the non-protonated histidine site with the asparagine residue (H0-L-N, a shift of one amino acid residue for LRR-10 from L-H0-L). The protonated histidine site is available at pH 7 but not at pH 9. The change from above −kT to below −kT predicts sufficient stability to be rarely reversible under standard conditions.

**Figure 4.**
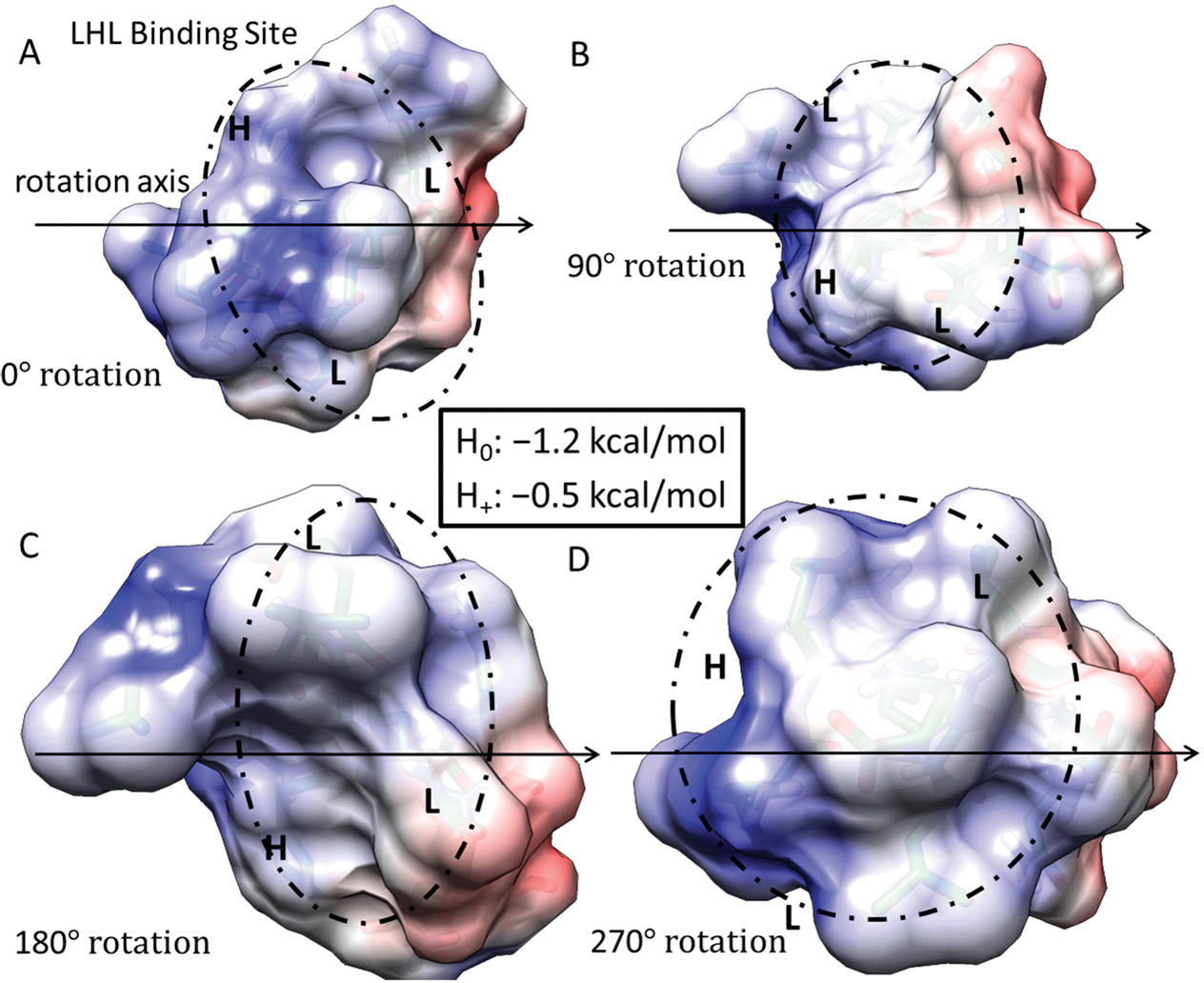
Free energy of binding estimates for sets of three adjacent residues for LRR-10 (LRELHLNNN) on human collagen type I alpha chain 2 (COL1A2) residues 861–871. The binding site (LHL) reference view is in (**A**), with views of 90° rotations along the indicated axis in (**B**–**D**). Each circle represents the same potential three-amino-acid binding site between the LRR-10 peptide and collagen at different rotations.

We see that the LHL interaction between LRR-10 and COL1A2 is below −kT when the surrounding environment is non-acidic or alkaline, meaning it is predicted to be unlikely to reverse under standard conditions. The prediction denotes that the favorable free energy necessary to couple for the reverse reaction of unbinding to happen is not likely to occur under standard conditions. LRR-10 also has likely interactions with collagen when the histidine is protonated. The shift in the binding free energy moves the binding site to above −kT, predicting that the binding is more likely to be reversible. When the histidine is protonated, other nearby binding sites of LRR10 may lead to more resonance structures which are close in energy, leading to rearrangements of LRR10 on the surface, with interactions of L, R+, E− and G, P, Q being favored the same as the L, H+, L interaction to within a difference of about 0.2 kcal/mol. Both of the potential binding sites for LRR-10 are shown in Figure 4.

**Figure 5.**
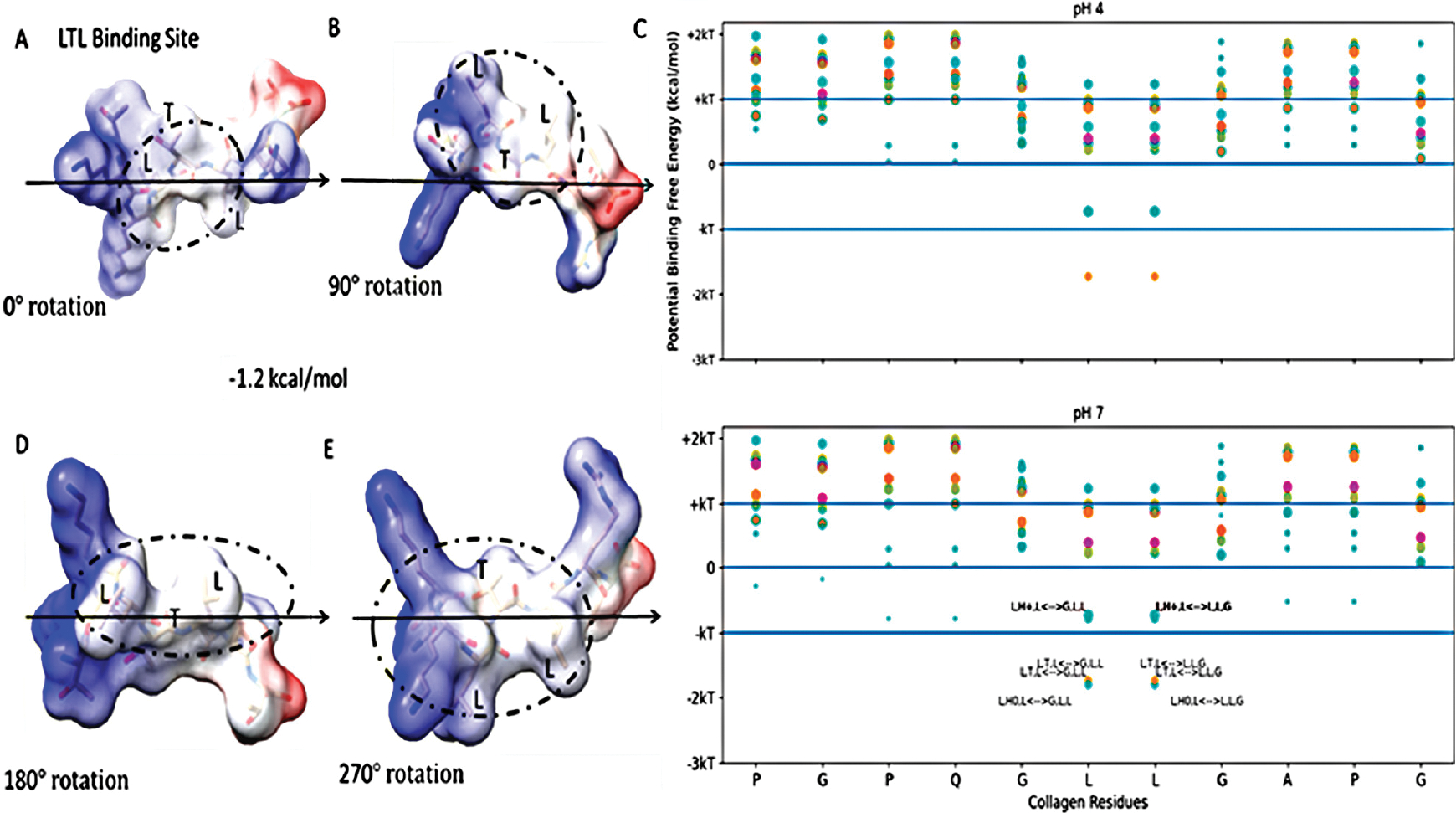
Free energy of binding estimates for sets of three adjacent residues for the high-stability collagen-binding peptide (TKKLTLRT) on human collagen type I alpha chain 2 (COL1A2) residues 861–871. The binding site (LTL) reference view is in (**A**), with views of 90° rotations along the indicated axis in (**B**,**D**,**E**). Each circle represents a potential three-amino-acid binding site between a peptide and collagen. Positive binding sites are energetically unfavorable, while negative binding sites are favorable. Binding sites significantly below −kT are likely to be stable. The potential binding free energy was calculated from the Wimley–White hydropathy scale values for the amino acids within the binding site. In (**C**), the predicted binding sites for the TKKLTLRT peptide (orange) was shown to include a binding site (LTL of peptide to GLL of collagen or LLG of collagen) at pH 4 which is missing from TKKTLRT (magenta). The LRR-10 cyan labeled binding sites are the same as in Figure 3. This binding site is the lowest free energy binding site among LRR-10 binding sites (cyan) and TKKTLRT binding sites at pH 4. This labeled TKKLTLRT binding site of LTL at pH 7 is about the same free energy as the lowest energy binding site of LRR-10 shown in Figure 4.

How the rearrangement of LRR-10 when bound to collagen based on protonation state affects its binding is an open question beyond our single-site binding model. However, our binding models indicate that the histidine residue in the leucine-rich repeat motif seems to serve as a logical switch determining if the hydropathic feature is a match for this hydropathic interface of COL1A2. If we consider the case when the histidine residue is replaced by a polar, uncharged amino acid like threonine, we can propose a proton-independent type of binding site for COL1A2 matching the hydropathic interface at this site. We describe this peptide sequence (TKKLTLRT) as a high-stability binding peptide because the collagen-binding prediction is nearly completely pH independent. See Figure 5 for the LTL binding site within the TKKLTLRT structure. We introduced the LTL peptide sequence from the previous peptide to result in TKK-LTL-RT. We compared TKK-LTL-RT to LREN-LTL-NNN to see which candidate high-stability collagen-binding peptide has the more favorable binding prediction to COL1A2. In Figure S2, we show that TKK-LTL-RT is predicted to have more favorable energy of binding than LREN-LTL-NNN. We inserted a leucine in the fourth amino acid position, thus providing the L-T-L binding site from LRR-10 with the histidine substitution within the TKKTLRT motif. Merging the hydropathic feature from LRR-10 to TKKTLRT created a predicted high-stability binding peptide that is almost completely pH independent while expanding the collagen-binding capability of the peptide across sites accessible to both original motifs.

### 3.2. Potential Binding Sites for Collagen Type I Alpha Chain 2

**Figure 6.**
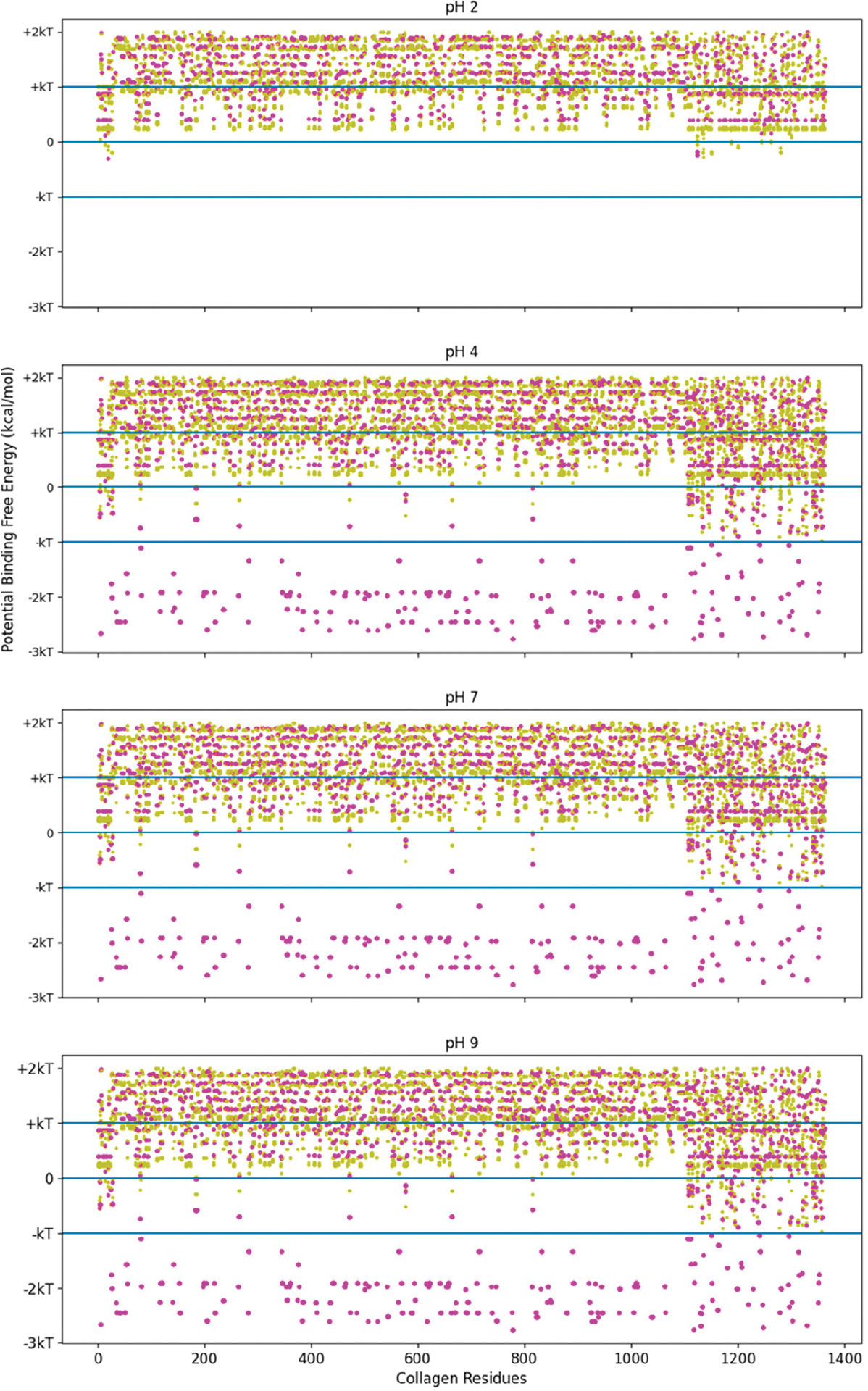
Free energy of binding estimates for sets of three adjacent residues for three different collagen-binding peptides for all residues of human collagen type I alpha chain 2 (COL1A2). Positive binding sites are energetically unfavorable, while negative binding sites are favorable. Binding sites significantly below −kT are likely to be stable. Each circle represents a potential three-amino-acid binding site between a peptide and collagen. The potential binding free energy was calculated from the Wimley–White hydropathy scale values for the amino acids within the binding site. The potential binding sites are magenta for COL1A2 Sense Peptide (TKKTLRT) and gold for collagenase sub-sequence (SQNPVQP). While neither peptide sequence has amino acids which a change state at different pH levels, the COL1A2 does have residues which are pH dependent. At pH 2, only E0 and D0 residue states are available. The alkaline residues of TKKLRT cannot interact with these uncharged residues, nor can the protonated histidine residues. At pH 4 and more alkaline pH values, the E0 and D0 residues can become charged, resulting in many strong interactions along the COL1A2 sequence and the alkaline side chains of TKKTLRT. kT, which is the Boltzmann’s constant multiplied by the temperature of 25 °C, is 0.693 kcal/mol. This value is close to a mean estimate of the free energy fluctuations of a surrounding environment near room temperature.

The high-stability collagen-binding peptide’s predicted interactions covered more of the pH range than with TKKTLRT alone. To see this trend, we investigated the screening method’s binding interactions across all of the residues of COL1A2. This analysis was a first approximation that did not include the hydropathy change from proline to hydroxyproline, which is more electronegative with the addition of the hydroxyl group. Because proline is an electropositive amino acid before the addition, the hydropathy is closer to octanol than to water after the substitution. Hydrophobic interactions are more likely than they appear in our screening method. See Figure 6 for the likelihood of binding for the TKKTLRT (magenta) and SQNPVQP (gold) peptides across the COL1A2 residues.

**Figure 7.**
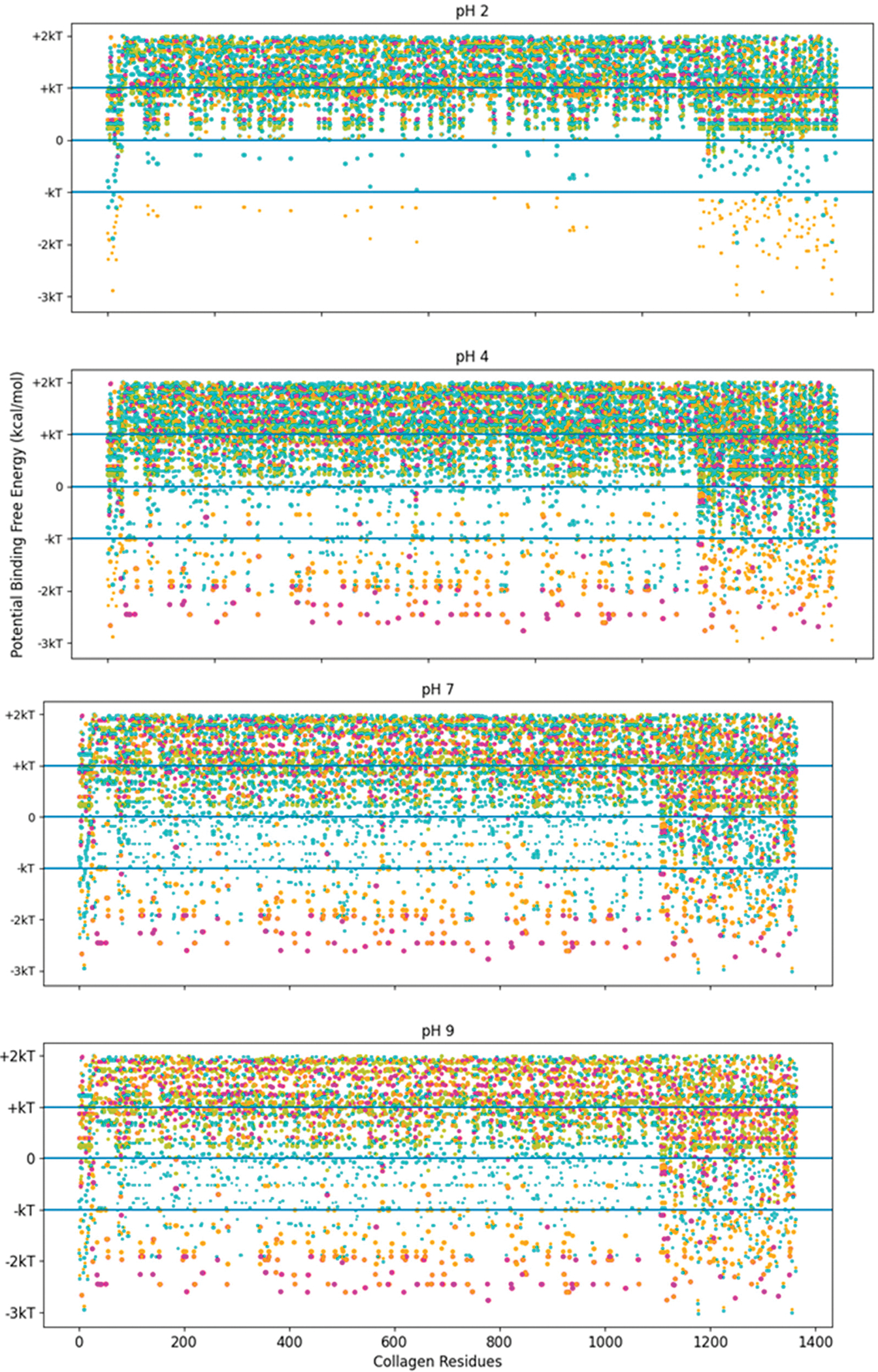
Free energy of binding estimates for sets of three adjacent residues for three different collagen-binding peptides for all residues of human collagen type I alpha chain 2 (COL1A2). Positive binding sites are energetically unfavorable, while negative binding sites are favorable. Binding sites significantly below −kT are likely to be stable. Each circle represents a potential three-amino-acid binding site between a peptide and collagen. The radius of the circle sizes is related to the magnitude of the polarity of the peptide residues. The potential binding free energy was calculated from the Wimley–White hydropathy scale values for the amino acids within the binding site. The potential binding sites are cyan for LRR-10 (LRELHLNNN), gold for the collagenase sub-sequence (SQNPVQP), magenta for the COLA2 sense peptide (TKKTLRT), and orange for the designed peptide (TKKLTLRT). The inserted leucine gave the peptide analogue a binding site with more potential binding free energy than LRR-10. TKKLTLRT is also not dependent on any of its residues changing protonation state to achieve this binding free energy. kT, which is the Boltzmann’s constant multiplied by the temperature of 25 °C, is 0.693 kcal/mol.

The pH independence of the high-stability collagen-binding peptides comes from its ability to interact more readily with hydrophobic interfaces like LRR-10 (See Figure 7). The inserted leucine gives the designed peptide a binding site with similar potential binding free energy to LRR-10, which is achieved without depending on the pH state of any of its residues. We had some predicted binding sites for TKKLTLRT at pH 2, and we had no predicted binding sites for TKKTLRT at pH 2. The TKKLTLRT still retains the strong predicted binding with COL1A2 sub-sequences with D and/or E amino acids, which are up to 1 kcal/mol more favorable than the LRR-10 hydrophobic binding sites. The increase in potential energy of binding comes from the difference in hydrophobic character of protonated histidine from the LRR-10 interface and hydrophobic character of replacing histidine with threonine from the peptide analogue interface at pH 2. This difference implies that the binding sites between COL1A2 and the peptide analogue are not likely to be reversed, whereas the LRR-10 binding sites are likely to be reversible as histidine changes protonation states. At more alkaline pH values, both peptides have binding sites which are not likely reversible. The exception is that LRR-10 binding sites are reversible by protonation of its histidine.

The trends of Figure 7 interactions may also apply to other extracellular matrix proteins. The potential binding sites of these peptides were evaluated for decorin, the protein origin of LRR-10, in Figures S3 and S4. First, we showed that collagenase binding peptide does not have strong electrostatic interactions with decorin, which is similar to COL1A2. The peptide sequence lacks charged residues, so strong electrostatic interactions with the collagenase peptide are lacking. For TKKTLRT and TKKLTLRT, electrostatic interactions are possible. We also highlighted an important characteristic of COL1A2, which is that the triple helix structure increases the availability of residues compared to other protein tertiary or quaternary structures. The LTL motif had many predicted hydrophobic interactions with decorin. However, due to energy landscape minimization constraints, hydrophobic interactions are less likely to be available depending on the strength of hydrophobic interactions. Therefore, the interacting partners for these interactions are likely to be interior for most proteins. However, if proteins are unfolded, then these interactions may become available. Similar to the LTL motif in the high-stability collagen-binding peptide, the LHL motif in LRR-10 was predicted to have many strong hydrophobic interactions with decorin if those sites are available.

### 3.3. Experimental Validation of Predicted Peptide

To examine if the insertion of the leucine in TKKTLRT results in more binding coverage, we synthesized both peptides, i.e., TKKTLRT and TKKLTLRT, with a biotin tag. The biotinylated peptides were next mixed with Cd/ZnS quantum dots (Q-dots) coated with streptavidin with a red emission maximum of ~655 nm. High binding affinity of streptavidin for biotin results in the formation of very stable peptide-coated Q-dots.

**Figure 8.**
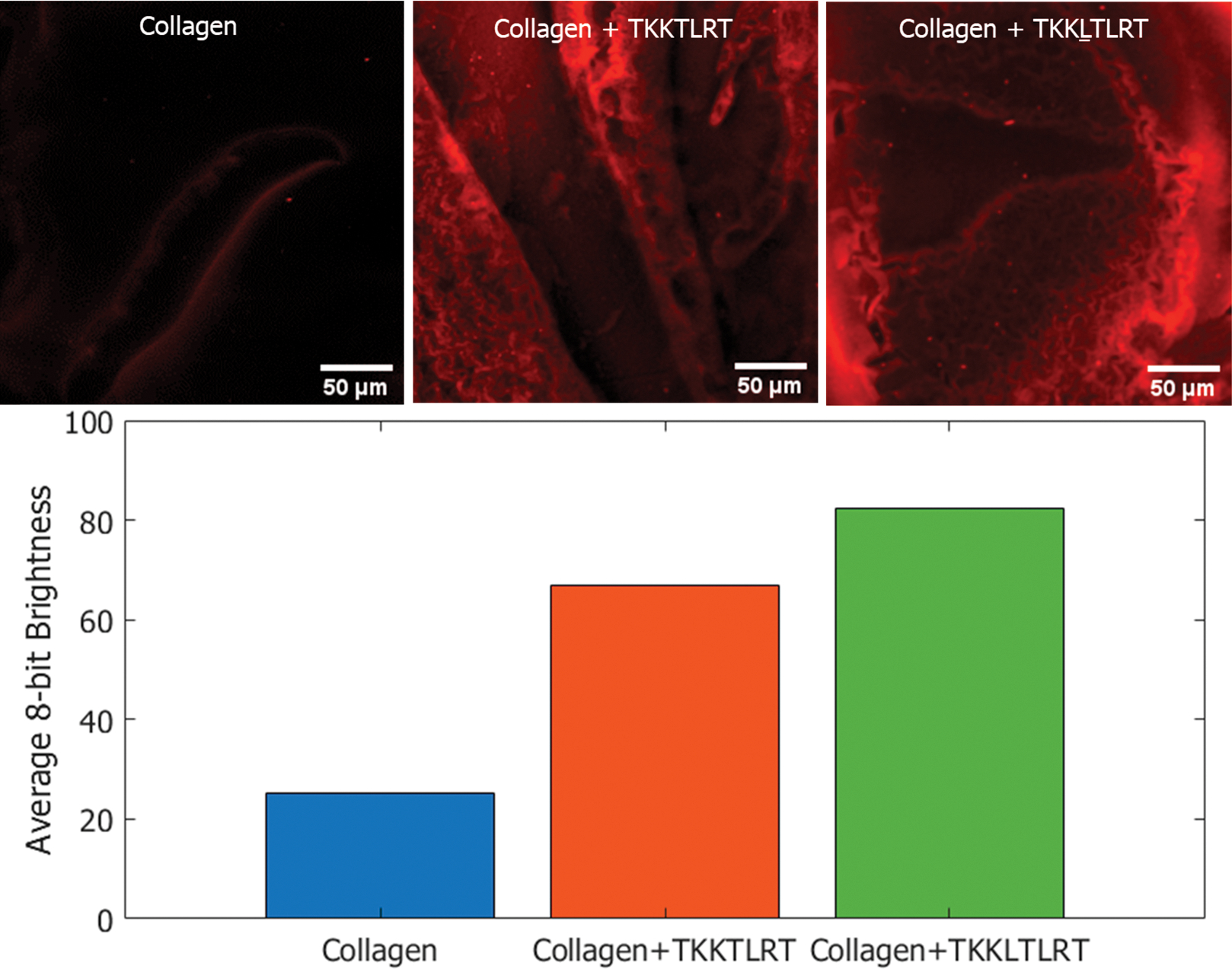
Streptavidin-coated quantum dot labelling of biotinylated peptides on type I collagen. The Q-dots complexed with biotinylated peptides remained on collagen after washing. The corresponding summed pixel intensities are provided in the bar chart. The predicted peptide TKKLTLRT resulted in the highest coverage based on the quantum dots assay.

We incubated the labeled peptides with type I collagen and washed the samples. As a negative control, collagen was incubated with streptavidin quantum dots without biotinylated peptides. Confocal microscopic images of the peptide-coated Q-dots and control samples are provided in Figure 8. Q-dot fluorescence was captured as a Z-stack with an excitation wavelength of 405 nm and emission wavelength of 655 nm (643 nm to 670 nm excitation range was evaluated). Images were processed using ImageJ 1.53 t. The bar plot in Figure 8 displays the averaged pixel intensities of the labeled images, with 255 being the maximum brightness for a pixel. The Q-dots showed some degree of non-specific binding affinity to collagen. However, among the two peptides tested, the predicted peptide TKKLTLRT showed higher coverage and the largest sum of pixel brightness as shown in the Figure 8 bar plot.

Building upon the hydropathy interface model we described, we hypothesize that these collagen interfaces are highly related to the binding sites of collagen of the leucine-rich repeat motifs. Previous studies have shown that TKKTLRT binds to type I collagen. Using our design, we increased binding of the peptide to collagen and validated it by streptavidin-coated quantum dots. The increase in collagen binding is likely related to the collagenase peptide SQNPVQP binding sites, for which TKKTLRT shares hydropathic features. The binding of the TKKLTLRT peptide indicates the possible union between SQNPVQP-related binding sites and the LRR binding sites. The engineered TKKLTLRT peptide is predicted to have stable binding at these sites under a wider variety of pH conditions than the LRR-10 peptide at its binding sites. This prediction is limited to single binding sites. Further studies are required to determine if the cooperative effect of multiple binding sites from LRR-10 favor the LRR-10 binding over the TKKLTLRT peptide on collagen.

## 4. Discussion

De Souza and Brentani observed that the DNA/mRNA complementary sequence (A- > T, T- > A, G- > C, C- > G) can be a viable source of amino acid sequences which bind to the collagen type I protein [36]. Their study demonstrated the complementary residues 772–778 for the COL1A2 gene, which encodes collagen type I alpha unit 2 in humans, is a peptide which binds to collagen type I. The collagen amino acid sequence is GPQGLLG. The complementary amino acid sequence is TKKTLRT. The authors then compared the hydropathy trend of TKKTLRT with SSNTLRS (analogous sequence in mouse) and SQNPVQP (fibroblast collagenase sequence) using the Kyte–Doolittle hydropathy scale, and they showed a strong similarity of profiles. In studying the hydropathy plots of COL1A2 sense peptide (TKKTLRT), collagenase peptide (SQNPVQP), and collagen type I chain α_2_ (GPQGLLG), we noticed trends in the hydropathy curves between the sequences. The TKKTLRT and SQNPVQP have similar trends in hydropathy plots in the Wimley–White scale and nearly identical hydropathy plots for the Kyte–Doolittle scale. The de Souza paper showed a similar strength of interaction between collagen and TKKTLRT and SQNPVQP through radioactively labeling collagen and quantifying the dose-dependent inhibition of gamma radiation for each peptide.

Studies have explored sense–antisense peptide interactions in many protein contexts. In HIV research, Brown et al. recognized the sense–antisense interactions that underlie the relationship between HIV glycoproteins and the host cell HIV receptors [43]. This hypothesis has also been tested for a ribonuclease S-peptide using analytical chromatographic affinity experiments, which concluded that there is evidence for antisense peptide binding [44]. A recent review estimates that such peptide pairings have been experimentally confirmed in more than 50 separate systems [34]. Recent progress has also shown evidence that tRNA, which pair with mRNA in the translation process, holds structural information about the attached amino acid that is additional to the standard codon table [45]. New models of the information contained within the standard genetic code through previously unstudied symmetries have recently been proposed [46–48]. While it is unclear how much information the biological machinery contains, the method of identifying generative relationships between nucleic acid sequences and protein sequences can be applied to the small-codon-like segments of peptides and proteins to begin to see rules which describe the binding properties of peptide and protein sequences. To evaluate this hypothesis further in the context of collagen-binding peptides, we developed a screening method using hydropathy for identifying likely binding sites for collagen using three consecutive amino acid segments, in parallel to codons within nucleic acid sequences.

Collagen-binding peptides hold promise for advanced technologies in materials science and in medical treatments. A recent study has shown that collagen-binding peptides can be molecular-level markers for mechanical damage in collagen, which is applicable to assessing the integrity of collagen within a system [49]. The mechanical properties of collagen are not just being characterized by computational approaches. Much progress is being made in understanding the toughening mechanisms in collagen systems and how to build bioinspired materials with bottom-up approaches [50,51]. The interfacial challenges in these materials are often multi-dimensional and interdependent on several environmental factors. These interdependencies make the design of material interfaces within collagen systems suitable for advanced neural networks which simultaneously consider interdependencies at multiple scales [52].

The TKKTLRT motif has been shown to create functional interfaces as a fusion tag in multiple studies [53–57]. Collagen–peptide interfaces are central to many different approaches related to collagen treatments and for extracellular matrix components for therapeutics. We review a few of these approaches, acknowledging that many other approaches are currently being investigated. A recent paper for guided bone regeneration has shown successful chimeric peptides combining two different collagen-binding peptides, TKKTLRT for binding to collagen type I and KELNLVY for binding to collagen type III, to bring antibacterial and healing properties to small intestinal submucosa (SIS) tissues [54]. The combination of TKKTLRT with other polypeptide sequences also extends to growth factors. A higher-stability version of TKKTLRT may lead to the opportunity to achieve the same effect with a smaller amount of growth factor or slower release kinetics to allow for a more sustained release of growth factor over a longer period of time.

Another recent study mitigating liver fibrosis in mice used TKKTLRT (CBD) attached to vascular endothelial growth factor (VEGF) to show reduced liver inflammation and fibrosis. The treated mice were injected with CCl_4_ at levels known to be toxic to the liver. The delivery of VEGF through the collagen-binding domain allowed for higher effective concentrations of VEGF compared to injecting it alone. The study concluded that CBD-VEGF resulted in increased vascularity, liver fibrotic attenuation, and hepatocyte apoptosis reduction. In prior studies, Dai et al. showed brain-derived neutrophic factor, nerve growth factor β, and basic fibroblast growth become targeted with slowed diffusion and increased retention of the GFs in the tissue with the peptide tag. These increased retentions led to enhanced wound repair and tissue regeneration [57]. CBD was found to increase the delivery of the GF compared to WREPSFCALS, which is derived from von Willeband’s factor. This increase in delivery has been attributed to the increase in polar residues of TKKTLRT compared to WREPSFCALS [58]. Again, we propose that a higher-stability binding peptide to pair with VEGF would lead to either further enhancement of repair and tissue regeneration or a smaller amount of functionalized VEGF to achieve the same therapeutic outcome. We hypothesize that the arrangement of a peptide’s residues in addition to their polarity is critical to its collagen-binding properties. We quantify this relationship through our screening method.

Designing tailored interactions with collagen under a variety of conditions will benefit several therapeutic applications. In Figure S5, we demonstrate layering the estimates of the hydropathy free energy with the conformational constraints of a solved structure for decorin [59–61]. Accessibility layering shifts binding energies of inaccessible residues towards zero. Mapping the fragments that are most likely to interact provides a guidance for further binding conformational analysis to be the most effective. How the binding events between proteins and peptides may affect the protein structure is an example of further computational analysis. Free energy binding estimation is shown to predict modifications on the collagen-binding peptide TKKTLRT that has led to improved binding to collagen type I under physiological pH conditions. Future studies using our approach could also provide the predictions of the high-stability analogue sequences for pH values less than 4 and enable the use of short peptides in several different application areas.

## 5. Conclusions

In this study, we developed a screening tool to highlight hydropathy free energy features of three different collagen-binding peptides to rationally engineer a high-stability collagen-binding peptide. We used sequence features from two collagen-binding peptides to rationally engineer a collagen-binding peptide with enhanced binding property. We extended the design of sense/antisense peptides for collagen-binding applications. We provided a hydropathy-based screening method which is pH dependent for peptide–collagen interactions, which is designed to reduce the computational time of virtual screening by reducing the number of molecular docking attempts for false binding cases. Furthermore, we used this method to show the design of a collagen-binding peptide with predicted high stability of binding across a wide range of pH environments. This hydropathy-based strategy is useful for screening peptide designs with less computational effort than molecular docking all interacting candidate subsequences, and it has a low false negative rate. Our scanning method assumes all collagen and peptide residues are available at the surface. This assumption helps to avoid false-negative binding results and allows for the inclusion of surface availability filtering as a separate tool. If the screening calculation time reduces the molecular docking computation time more than the screening calculation time, computational screening will be faster and more accurate. If the screening computational time is less than the reduction of molecular docking computation for true negatives the screening method identifies, it saves computational time to be invested in other virtual screening methods. Therefore, this strategy can serve as an enrichment tool for virtual screening processes where molecular docking can lower the false-positive rate of the design candidates. Virtual screening processes such as AutoDock [62] and structure-based virtual screening (SBVS) approaches [63] would benefit from more focused molecular docking so that more design features can be considered for drug candidates with the same computational resources.

## Supplementary Material

Supplementary file

## Data Availability

Not applicable.
