# Supplementary material for: Designing Collagen-Binding Peptide with Enhanced Properties Using Hydropathic Free Energy Predictions
Source: Appl Sci (Basel). Author manuscript; Available in PMC 2024 Mar 6. (PMC10686322; doi:10.3390/app13053342)
Supplement: Supplementary file [file NIHMS1894486-supplement-Supplementary_file.pdf]

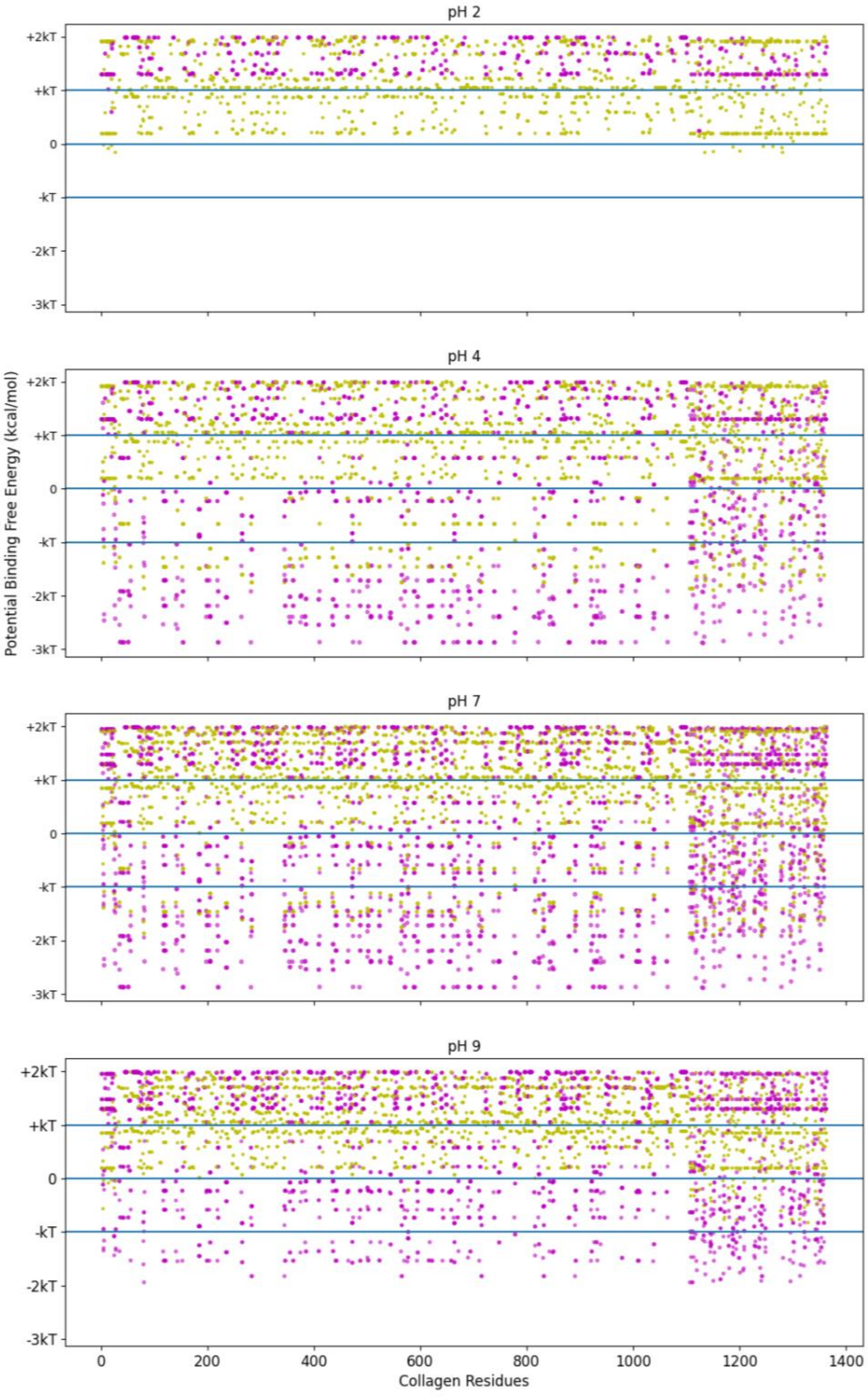

Figure S1. Evaluation of hydropathic free energy estimates for collagen III-derived peptide examples. GLKGHR was shown to bind to collagen type I in Figure 1F of Kalamjski *et al.*[1]. GLAGHA binds to collagen type I significantly less than GLKGHR. We show the predicted binding site free-energies of these two peptides for collagen type I alpha chain 2 (COL1A2). GLKGHR (magenta) has more potential binding sites which are more energetically favorable than -kT than GLAGHA (gold) at pH 7. Positive binding sites are

energetically unfavorable while negative binding sites are favorable. Binding sites significantly below –kT are likely to be stable. Our method predicts less binding for GLAGHA at pH 9 and pH 2 than at pH 4 and pH 7. An environmental pH 9 would provide the largest likelihood of specificity of binding for GLKGHR compared to GLAGHA. Our model does predict some binding for GLAGHA at pH 4 and pH 7. The procedure for stopping the binding in the Kalamjski *et al* study was to add 2 M sulfuric acid to rapidly lower the pH. Our free energies of binding agree that both peptides do not have predicted binding sites below –kT at pH 2. The larger quantity of binding sites below -kT from pH 4 – pH 9 for GLKGHR compared to GLAGHA, as well as the lower energy of these sites is consistent with GLKGHR having measurably better binding performance except at pH 2 or lower. As the pH increases, the interfaces between the collagen and peptides decrease in the number of protons. This charge gradient affects both the collagen type I hydropathic free-energies and the considered peptide hydropathic free energies.

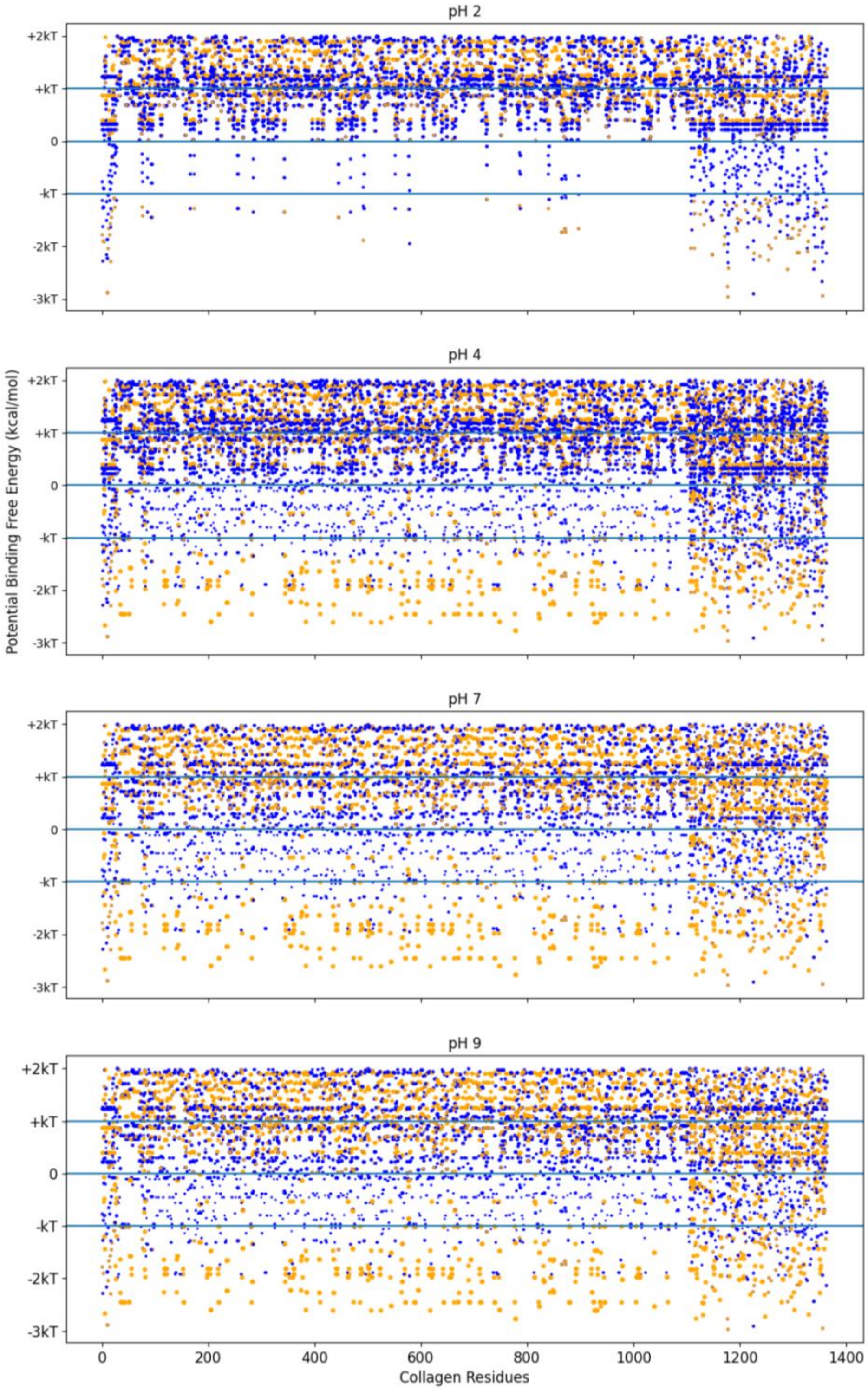

Figure S2. Evaluation of hydropathic free energy estimates for candidate high-stability binding peptides TKKLTLRT (orange) and LRELTLNNN (blue). We show the predicted binding site free-energies of these two peptides for collagen type I alpha chain 2 (COL1A2). Positive binding sites are energetically unfavorable while negative binding sites are favorable. Binding sites significantly below –kT are likely to be stable. Our method predicts more energetically favorable binding sites for TKKLTLRT (-3kT) binding to COL1A2 compared to LRELTLNNN binding to COL1A2 (–2kT).

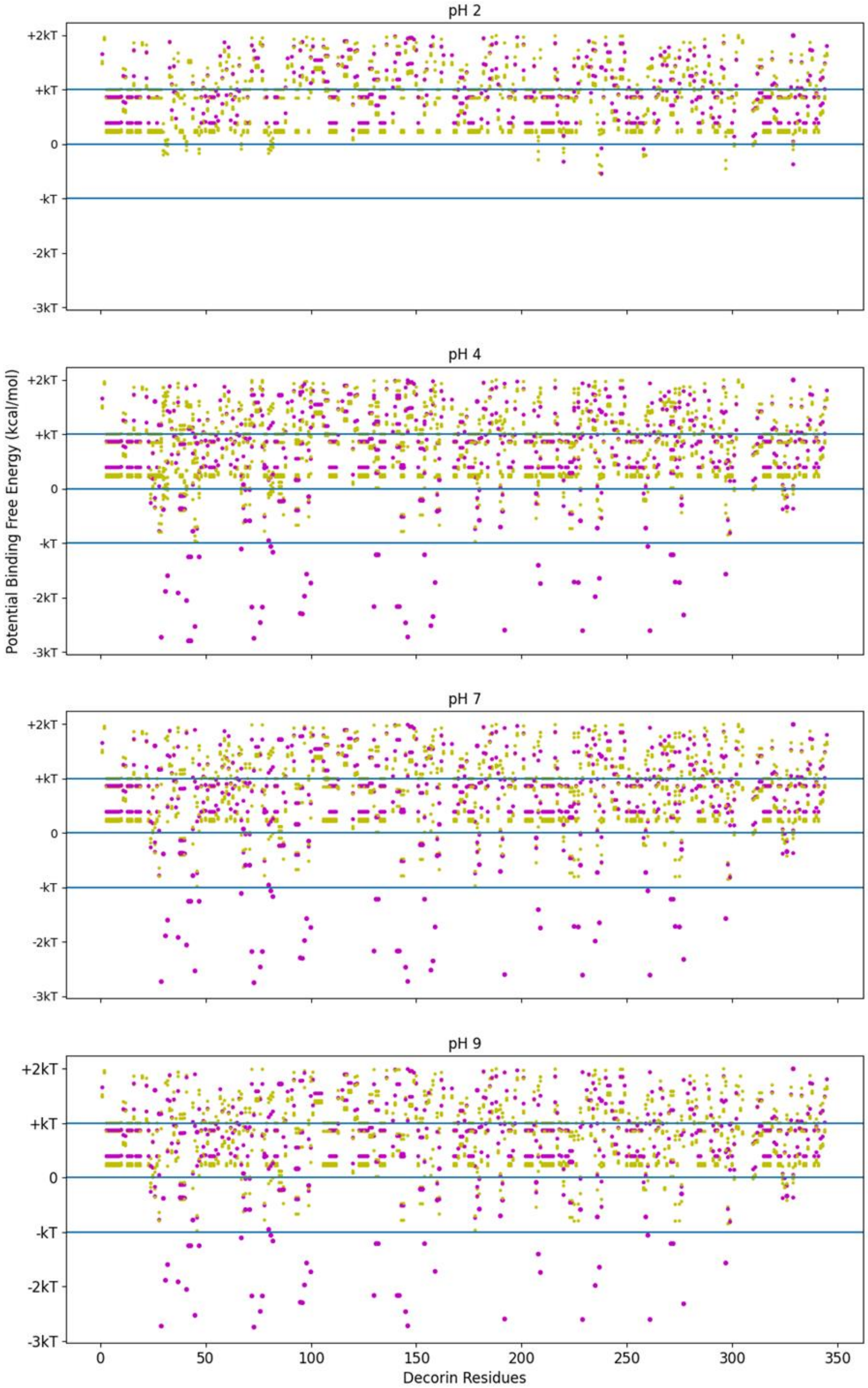

Figure S3. Evaluation of collagenase peptide (SQNPVQP -- gold) and COL1A2 DNA complement peptide (TKKTLRT -- magenta) on decorin. Positive binding sites are energetically unfavorable while negative binding sites are favorable. Binding sites significantly below –kT are likely to be stable. To investigate how the trend from COL1A2 predicted binding sites compares to a different protein, we chose decorin. At pH 2, the large availability of protons increases the free energy of binding for both peptides, though no charge state of the peptides are changed. The decorin protein, like COL1A2 has reduced charged interactions

above pH 2 by our hydropathy screening method. At pH 4 and pH 7 more proton-accepting residues are not saturated and become potential binding sites. For these potential binding sites to be available, they must be at the solvent accessible surface for decorin. Some of these sites may only be available when decorin is unfolded or misfolded. COL1A2 has a secondary, tertiary and quaternary helix structures which makes the majority of residues in its chain available at the surface. For most proteins, especially globular proteins, this accessibility is only for a minority of residues. If completely unfolded, the TKKTLRT peptide has more binding sites and more favorable binding sites than SQNPVQP.

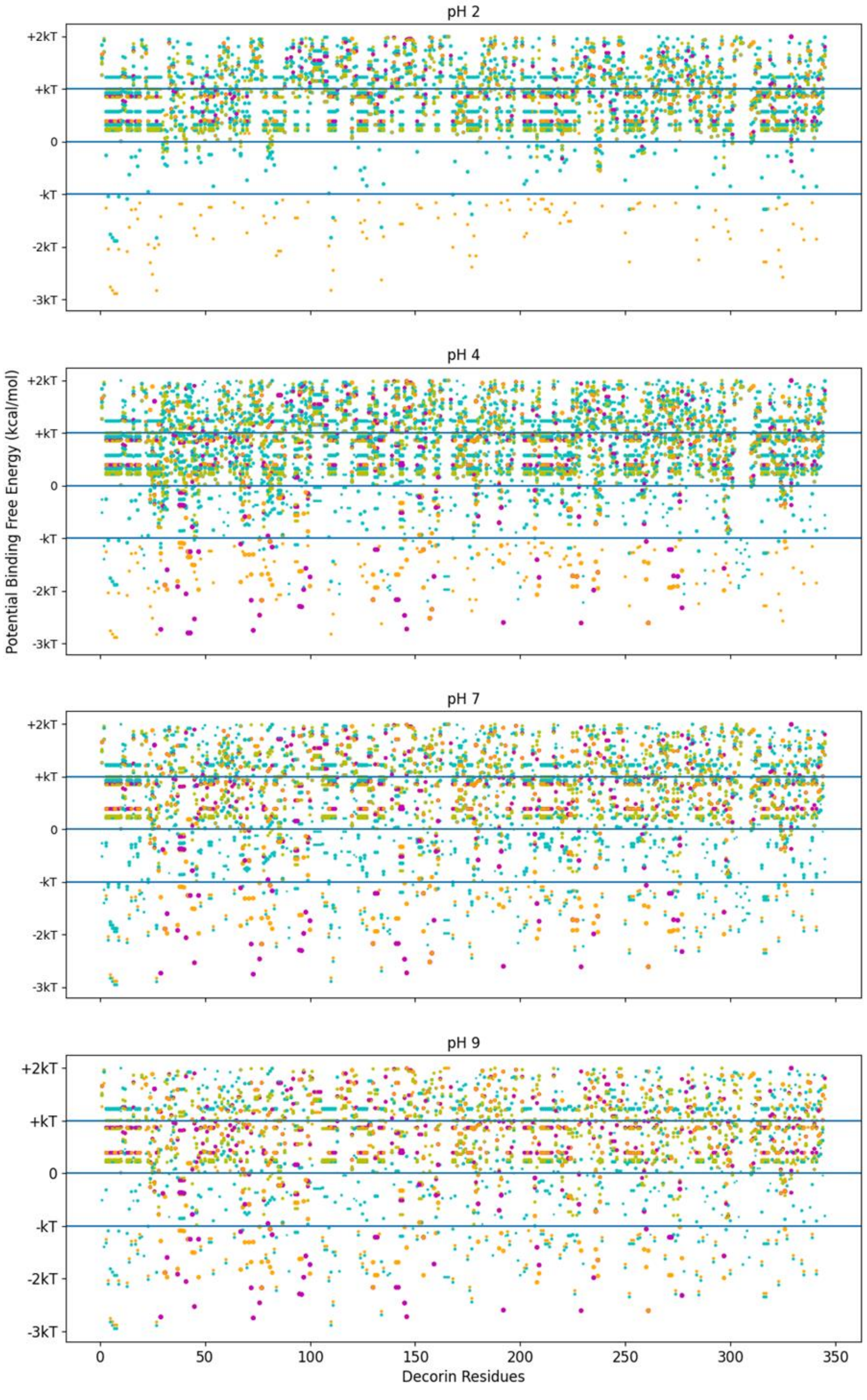

Figure S4. Evaluation of collagenase peptide (SQNPVQP -- gold), COL1A2 DNA complement peptide (TKKTLRT -- magenta), high-stability collagen-binding peptide (TKKLTLRT -- orange) and LRR-10 (LRELHLNNN – cyan) on decorin. The side of the binding markers is proportional to the polarity of the interaction. More hydrophilic interactions have larger markers. We extend the evaluation of the potential

binding sites of the peptides from collagen Type I to decorin, another extracellular matrix protein. Positive binding sites are energetically unfavorable while negative binding sites are favorable. Binding sites significantly below –kT are likely to be stable. These potential binding sites must be available at the decorin surface to be applicable. The folding state of decorin determines this availability. If the decorin is completely unfolded, then all potential binding sites are available. Under this condition, the high-stability collagen peptide is predicted to have better binding to decorin at pH 2. The high-stability collagen binding peptide-decorin binding sites are available through hydrophobic interactions, noted by the small marker size. If decorin is folded such that hydrophobic interactions are on the interior, which is highly likely according to energy landscape properties, then the most hydrophobic sites will not be available. LRR-10 also has predicted binding sites which rely on hydrophobic interactions. If decorin is folding with hydrophobic interactions on the inside, then LRR-10 is not likely to have interactions with decorin. The polar residues of TKKTLRT and TKKLTRT are more likely to have non-specific binding interactions with decorin. It is unclear, however, that any binding non-specific interactions with decorin would result or would not result in any allosteric effects on the function of decorin.

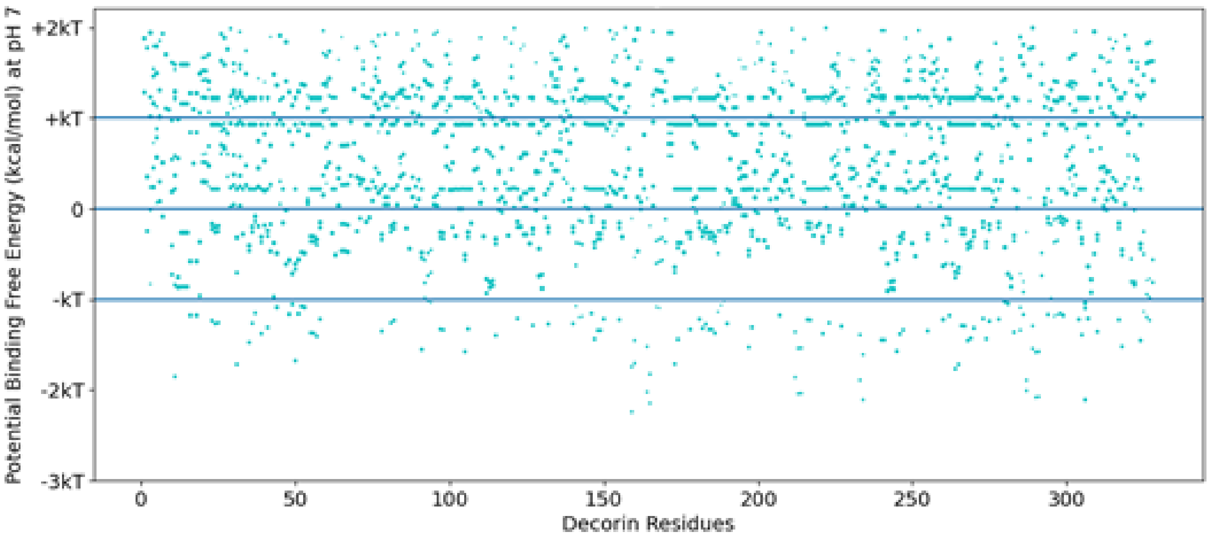

Figure S5. Evaluation of binding free energy between LRR-10 and decorin at pH 7 by accessibility of decorin residues. Decorin structure (PDB:1XKU) [2] measured with DSSP [3] for solvent accessibility (ACC) in square angstroms. Free-energy values normalized by the product of the ratio of the solvent area accessibility of each residue to the published solvent area estimates of each residue. [4] The potential binding interactions of LRR10 and decorin are shifted toward zero for hydrophobic residues of decorin in between the parallel beta-sheet interior concave surface and the outer concave surface of helical segments. Binding interactions between -2 kT and -3 kT for decorin residues 100-150 are shifted toward more reversible free-energy values, for example. Our hydropathy-based free-energy estimation tool can be stacked with layers of conformational information to improve binding energy estimates.

References

(1) Kalamajski, S.; Bihan, D.; Bonna, A.; Rubin, K.; Farndale, R. W. Fibromodulin interacts with collagen cross-linking sites and activates lysyl oxidase. *J. Biol. Chem.* **2016**, *291* (15), 7951-7960.
(2) Scott, P. G.; McEwan, P. A.; Dodd, C. M.; Bergmann, E. M.; Bishop, P. N.; Bella, J. Crystal structure of the dimeric protein core of decorin, the archetypal small leucine-rich repeat proteoglycan. *Proceedings of the National Academy of Sciences* **2004**, *101* (44), 15633-15638.
(3) Kabsch, W.; Sander, C. Dictionary of protein secondary structure: pattern recognition of hydrogen‐bonded and geometrical features. *Biopolymers: Original Research on Biomolecules* **1983**, *22* (12), 2577-2637.
(4) Topham, C. M.; Smith, J. C. Tri-peptide reference structures for the calculation of relative solvent accessible surface area in protein amino acid residues. *Computational Biology and Chemistry* **2015**, *54*, 33-43. DOI: https://doi.org/10.1016/j.compbiolchem.2014.11.007.
